# A Sanatorium for Consumption

**Published:** 1900-12-01

**Authors:** 


### The Institutional Workshop.

### A SANATORIUM FOR CONSUMPTION.

### HAILEY-ON-THE-CHILTERNS.

By Our Special Commissioner.

Something like a generation must have passed since the open-air treatment for consumption was begun in Germany; but the slow moving, and, perhaps, on the whole, safe conservation of English physicians prevented its general introduction into English practice until the last few years. Now there are at least twenty-three in the British Isles. We are not able to state the total number of beds contained in these sanatoria, but presumably we rather over-state it at 20 each, which estimate would give us a total of 460 beds, and as the average period required for treatment is not less than four months, we arrive at the conclusion that not more than 1,400 patients could be treated in any year—a number falling so far short of the requirements that more, many more, sanatoria are urgently needed, and what is more particularly urgent is the erection of a large sanatorium for those who could not afford more than a guinea a week.

The Hailey Sanatorium is situated on a plateau between three and four hundred feet above the sea-level. Beautiful views are obtained from the garden of the sanatorium, and if we ascend another two hundred feet to the wooded margin of the estate the prospect is very fine. The building itself, as such buildings always should be, is secluded and far distant from high roads or obnoxious neighbours. The house is an old one altered to suit its present purpose. A large verandah has been put up and thorough cross-ventilation has been obtained in nearly all of the bedrooms. The rooms are, of course, destitute of hangings or carpets or of anything that could harbour dust. The floors are covered with cork-carpet, periodically rubbed with carbolic oil; the walls are varnished, and everything done to secure a high degree of sanitation throughout the establishment. Ornamental shrubs and forest trees surround the house, but not too near to interfere with the free circulation of air. Placed near the trees are several châlets for sleeping in. These are all raised on stanchions well above the ground; are built of wood with an air space between the outer and inner walls, and as the walls are left open at the eaves the space is used as a powerful ventilator. Each wall, in fact, might be described as an extended Tobin's tube. There are two windows on each aspect of the châlet and a ventilator in each gable. The inner walls are highly varnished and the outer ones are painted. Some of these châlets have folding doors, so that if need be the bedsteads may be wheeled into the verandah of the châlet. The rooms can be warmed when the patient is dressing or undressing; and each châlet contains one bed only. It is difficult to imagine anything more suitable for the open-air treatment of phthisis than these châlets. Certainly no room in any house or sanatorium can equal them; and, moreover, a patient occupying one can have as much privacy as he wishes, or when he wants company he can join the other patients in the large verandah of the house. At Hailey these châlets could be multiplied almost indefinitely, as the estate comprises nearly seventy acres, and under shelter of the flanking wood there are many places where they could be put up, the immediate vicinity of the wood affording shady and sheltered walks. The subsoil is sand and gravel, and the water is obtained from wells about 130 feet deep. Hailey is in Oxfordshire, about 4½ miles from Goring on the Great Western main line, but it is only about 2 miles from Wallingford on the branch line.

Following the example of the German sanatoria, drugs are not much used in the treatment, it being Dr. Reinhardt's opinion that "no medicine at present known has any specific action as a cure for tuberculosis; but there are many drugs which assist in strengthening the system and thereby they may assist the cure." The sheet anchors here and elsewhere are absolutely pure air night and day, abundant food (without alcohol except in rare cases) sterilised milk, rest, and carefully-regulated exercise. To these may be added constant medical supervision, no worries and troubles, and freedom from the misplaced kindness and interference of relatives. The patients are weighed twice a week, and Dr. Reinhardt is not satisfied unless a gain of two pounds takes place weekly. It is generally greater than this; and on the occasion of our visit the average of the patients then in residence had been three pounds weekly.

It might be imagined that a fair amount of philosophy would be required to bear this life of routine for the four months and often longer required for treatment, but it would seem that a feeling of monotony is not often present. On the contrary, it was impossible not to be struck with the happy appearance of the patients; and it must be that the new patient will have the element of hope springing up in him as he looks on the other cases progressing towards recovery.

